# AGING AND FEELING CURIOUS: A TIME-SAMPLING STUDY

**DOI:** 10.1093/geroni/igz038.3090

**Published:** 2019-11-08

**Authors:** Li Chu, Helene H Fung

**Affiliations:** 1 The Chinese University of Hong Kong, Shatin, New Territories, Hong Kong; 2 Chinese University of Hong Kong, Hong Kong, Hong Kong

## Abstract

Curiosity is commonly defined as “the desire for new information and experience.” While curiosity has been associated with numerous positive outcomes (e.g., improved well-being, better cognitive performance and longer life expectancy, some studies suggested that curiosity declined with age. However, very few studies actually attempt to examine why curiosity may be lower among older adults. Moreover, scholars disagreed on “why” people feel curious. According to the dual process theory (Spielberger & Starr, 1994), curiosity is induced by optimal level of uncertainty and anxiety with the desire to reduce these aversive feelings. However, the personal growth facilitation model (Kashdan, Rose, & Fincham, 2002) posits that people are curious intrinsically for one’s own growth, which is associated with positive affects. Therefore, the present study aims to examine age differences in the affective profile of feeling curious by comparing the momentary affective experience of curiosity between younger and older adults. In this study, we conducted a 2-week time-sampling study with 78 younger adults (age 19-29) and 79 older adults (age 60-85) from Hong Kong. Multilevel modeling analyses demonstrated a positive relationship between curiosity and positive emotions for both younger (β=.29, p<.01) and older adults (β=.70, p<.01). Interestingly, anxiousness was positively associated with younger adults’ curiosity (β=.09, p=.01) but not for older adults (β=.06, p=.29). Our study supported both theories, but suggested that one may be more dominant among older adults. These findings have important implications for future interventions to reduce anxiousness to encourage older adults to keep an open-minded attitude towards novelties.

